# Comparison of the serum level of interleukin‐4 in patients with brucellosis and healthy controls

**DOI:** 10.1002/jcla.23267

**Published:** 2020-02-26

**Authors:** Peyman Eini, Mohammad Mahdi Majzoobi, Hamid Reza Ghasemi Basir, Zahra Moosavi, Abbas Moradi

**Affiliations:** ^1^ Brucellosis Research Center Hamadan University of Medical Sciences Hamadan Iran; ^2^ Department of Pathology School of medicine Hamadan University of Medical Sciences Hamadan Iran; ^3^ Hamadan University of Medical Sciences Hamadan Iran; ^4^ Department of Community Medicine Hamadan University of Medical Sciences Hamadan Iran

**Keywords:** brucellosis, IL‐4

## Abstract

**Introduction:**

Evaluation of cytokines such as interleukin‐4 (IL‐4) can be an important tool in examining immune responses to brucellosis. Also, determining the response rate to treatment is necessary for controlling and eradicating of disease. The review of previous studies reveals contradictory results that require further research in this regard. The aim of this study was to compare the serum level of IL‐4 in patients with brucellosis and healthy controls.

**Material and Methods:**

In this descriptive‐analytical study for comparison of two groups, a total of 165 participants, including 83 patients with brucellosis and 82 non‐infected people, were evaluated after matching of sex and age in Hamadan (northwest of Iran) in 2017 and the serum level of IL‐4 was compared by ELISA method. The collected data were analyzed by SPSS software version 21 at 95% significant level.

**Results:**

Mean of age in the case and control groups were 50.25 ± 16.01 and 43.26 ± 15.6 years, respectively. The serum levels of IL‐4 in the case and control groups were 1.42 ± 0.51 pg/mL and 1.31 ± 1.02 pg/mL, respectively. Based on the non‐parametric Mann‐Whitney test, the IL‐4 level was significantly higher in the case group, compared with the control (*P* < .001), but no statistically significant relationship was found between serum levels of IL‐4 with age, sex, and serologic titers of Wright and 2ME.

**Conclusion:**

In patients with brucellosis, the level of IL‐4 increases independently of the duration and severity of the disease, which indicates the role of this cytokine of immune system in this infectious disease.

## 1 INTRODUCTION

Brucellosis is one of the most common diseases in humans, which is transmitted directly or indirectly from infected animals. The pathogenic agent of brucellosis is a non‐motile, non‐capsular, Gram‐negative, and intracellular bacillus which can cause chronic zoonotic diseases in humans. Brucellosis in humans involves various organs including the digestive system, liver, nervous system, blood vessels, heart, skin, eyes, and joints, causing serious damage.(1, 2)

Despite the control of brucellosis in many worldwide countries, this disease remains endemic in the Mediterranean and Middle Eastern countries including Iran.(3, 4) The prevalence of brucellosis in Iran has been estimated at 0.5 to 19.9 per 100 000 people in different regions, and most of the isolates are *Brucella melitensis*.(5, 6)

Understanding of immune system mechanisms can be helpful in the management of infectious disease. Evidence suggests that both humoral and cellular immune systems contribute to the clearance of brucellosis infection.(7, 8)

*Brucella* strains survive in some cells, such as macrophages, and can be released into the reticuloendothelial system by mononuclear phagocytes.(7, 9) The host protection against *Brucella* strains is primarily dependent on a cellular immune system including CD^4+^ and CD^8+^ T‐cell lymphocytes, as well as antigen‐presenting cells, such as macrophages and dendritic cells.(1, 9) Cytokines are significant contributors to immunity and inflammation. Early in the course of infection, cytokines, such as interleukin‐12 (IL‐12), stimulate interferon gamma (IFN‐γ) production, which triggers the response of T‐helper‐1 (Th1) lymphocytes and activates macrophages. The activated macrophages can destroy intracellular microorganisms and clear the infection.

Tumor necrosis factor‐α (TNF‐α) is also produced early in the immune response and stimulates cytotoxic T lymphocytes, which are able to relative clearance of contaminated cells. However, the capacity of virulent *Brucella* to suppress the immune response of TNF‐α may explain the limited role of this cytokine in protection against infection. On the other hand, some inflammatory cytokines, including IL‐6 and IL‐10, reduce immune system protective response.(10) The results of some studies have shown that T‐helper‐1 (Th1) lymphocytes activation is responsible for immune system protection, while the response of T‐helper‐2 (Th2) lymphocytes is effective in exacerbation of the disease.(11, 12) It seems that in brucellosis, the balance between Th1‐ and Th2‐produced cytokines is associated with infection or disease resistance. Th1 is known to produce IFN‐γ, while Th2 is an IL‐4 producer. On the other hand, IL‐4 has inhibitory effects on the stimulation and regulation of humoral and cellular immunity.(1) IL‐4 stimulates the production of autocrine growth factors for the development of Th2 lymphocytes and differentiation of naive CD^4+^ T lymphocytes. It also controls macrophage activity opposed to IFN‐γ effect; therefore, it can inhibit the rapid cellular immune response against brucellosis. It should be noted that IFN‐γ is the main Th1‐derived cytokine, which plays a major role in the activation of macrophages and control of brucellosis infection.(13)

IL‐4 was discovered first in 1980. This cytokine is produced by basophils, mast cells, eosinophils, and Th2 lymphocytes. IL‐4 contributes to the regulation of cell division, gene expression, and prevention of apoptosis in many cells, such as lymphocytes, macrophages, epithelial cells, fibroblasts, and endothelial cells. Moreover, IL‐4, along with other cytokines (eg, IL‐10, IL‐13, and IL‐15), contributes to the differentiation and transformation of CD^4+^ lymphocytes into Th2 cells; it also inhibits Th1 and reduces the production of IFN‐γ by Th1 cells. Another role of IL‐4 is regulation of immunoglobulin class changes and production of IgE and IgG‐4 in B‐cells.(14)

In a study by Akbulut et al(15) investigating intracellular cytokines produced by Th1 and Th2 lymphocytes in brucellosis via flow cytometry, despite the lack of a significant difference in the level of CD^4+^‐IL^4+^ cells between the brucellosis and healthy controls, the levels of CD^3+^‐IL^4+^ cells in patients with brucellosis were lower than healthy subjects.

The review of previous studies reveals contradictory results that require further research in this regard. In the study by Ahmed et al, the levels of IL‐4, IL‐12, TNF‐α, and IFN‐γ were investigated in patients with brucellosis and healthy subjects. In this study, the serum levels of IL‐4 and TNF‐α were not detectable in patients, whereas IL‐12 and IFN‐γ were higher in patients with brucellosis compared to healthy subjects, suggesting the induction of Th1 cytokines in human brucellosis.(16) In addition, Galanakis et al(17) compared the level of IL‐4 in children with brucellosis and healthy controls in Greece; the results indicated the increased level of IL‐4 in both acute and chronic phases of the disease.

In a study by Demirdag et al, the serum levels of IFN‐γ, TNF‐α, and IL‐4 were measured in healthy subjects recruited as the control group and patients with brucellosis before and after treatment. The serum levels of IFN‐γ and TNF‐α increased in the brucellosis group compared with the controls, and a significant reduction was observed after treatment, but the serum level of IL‐4 was not significantly different between the control and patient groups and also after treatment.(13) Furthermore, Reza et al studied IFN‐γ and IL‐4 levels in patients with brucellosis before and after treatment in Iran. In their study, the levels of IFN‐γ and IL‐4 significantly decreased after treatment.(18)

Generally, evaluation of cytokine production can be an important tool in examining immune responses to stimuli, such as pathogens, vaccines, and safety challenges. Also, understanding the survival mechanisms of intracellular Brucella and determining the response rate to treatment are necessary for controlling and eradicating the disease. Therefore, measurement of the level of IL‐4 in people with Brucella infections and its comparison with healthy people can increase our knowledge about the reactions of the immune system to this infectious disease.

## 2 MATERIALS AND METHODS

In this descriptive‐analytical study for comparison of two groups, patients with a diagnosis of brucellosis, who were admitted to the infection ward of Sina Hospital or the outpatient clinic, were recruited using the available sampling method. The inclusion criteria were a Wright test titer ≥ 1/80 and a 2ME test titer ≥ 1/40 in symptomatic patients. Written consent forms were obtained from all the participants, and their demographic characteristics were recorded. The blood samples (5 cc) were taken before starting the treatment, and the sera were stored at −70°C in the hospital laboratory until further analysis.

After reaching the desired number of samples, the serum level of IL‐4 was measured using the Human IL‐4 High‐Sensitivity ELISA Kit (Invitrogen), with a sensitivity of 0.1 pg/mL, assay range of 0.25‐16.0 pg/mL, and intra‐assay CV of 8.8%, based on the ELISA method using a Stat Fax 3200 ELISA reader (wavelength, 450 nm). The control group was selected after matching for age and sex. The history of healthy participants, who were referred to the laboratory for checkups, was also taken for evaluation of exclusion criteria. Blood samples were collected from these participants similar to the patients for further tests.

Moreover, the history of collagen vascular disease, anti‐inflammatory or corticosteroid drugs consumption, immunodeficiency, use of immunosuppressive drugs, prior antibiotic therapy that was effective for brucellosis, and recent history of infectious diseases other than *Brucella* were considered as the exclusion criteria in the both groups. Patients with complications of brucellosis (such as encephalitis and orchitis) were also excluded due to the probable potential effect of complications on the serum level of IL‐4. It should be noted that the person who performed the tests was completely blind to the group of subjects being tested.

The sample size was estimated at 45 subjects per group, based on the results of a study by Ahmed et al(16) on the evaluation of IL‐4 in patients with brucellosis and healthy subjects, using the sample size formula for detecting a difference between two populations, at 95% confidence level and statistical power of 90%. However, in order to increase the study power, 82 subjects were enrolled in each group.

In this study, SPSS version 21 was used to analyze the data. Descriptive data are presented in tables and charts by measuring central indices and distribution patterns by calculating ratios and percentages. In order to compare the mean serum level of IL‐4 between the two groups, Mann‐Whitney test was used (based on the results of Kolmogorov‐Smirnov test). Also, Student's *t* test was performed to compare the serum level of IL‐4 in patients. On the other hand, Spearman's correlation coefficient test was used for comparisons in terms of age. All statistical analyses were performed at 95% confidence level.

This study was approved by Hamadan University of Medical Sciences (research code, IR.UMSHA.REC.1396.792). All therapeutic interventions were routinely performed, and the tests were covered by the project funding. Data were collected without documenting any personal data, and the results were reported in general.

## 3 RESULTS

In this study, a total of 165 participants, including 83 patients with brucellosis (case group) and 82 healthy people without brucellosis (control group), were evaluated. In terms of sex, the case group consisted of 49 (59%) men and 34 (41%) women, while the control group included 40 (48.8%) men and 42 (51.2%) women; there was no significant difference in terms of gender between the groups (*P* = .186). Also, the mean age of subjects in the case and control groups were 50.25 ± 16.01 and 43.26 ± 15.6 years, respectively; however, the difference between the groups was significant in terms of age (*P* < .001), despite the matching of decades of age.

The serum levels of IL‐4 in the case and control groups were 1.42 ± 0.51 pg/mL and 1.31 ± 1.02 pg/mL, respectively (Table 1). Based on the non‐parametric Mann‐Whitney test, the IL‐4 level was significantly higher in the case group, compared with the control group (*P* < .001).

**Table 1:** Serum levels of IL‐4 in patients with brucellosis and control group

Variable	Group	
	Case	Control
Number	83	82
Mean of IL‐4 (pg/mL)	1.42	1.31
SD	0.51	1.02
*P* value	*P* < .001	

According to the results, the serum levels of IL‐4 in men and women with brucellosis were 1.32 ± 0.27 pg/mL and 1.55 ± 0.72 pg/mL, respectively. Based on the results of Student's *t* test in patients with brucellosis, there was no significant difference between male and female patients regarding the serum level of IL‐4 (*P* = .089). Meanwhile, based on the results of Spearman's correlation test, there was no significant correlation between the age of patients with brucellosis and the serum level of IL‐4 (Spearman's *ρ* = .089, *P* = .422).

**Figure 1:**
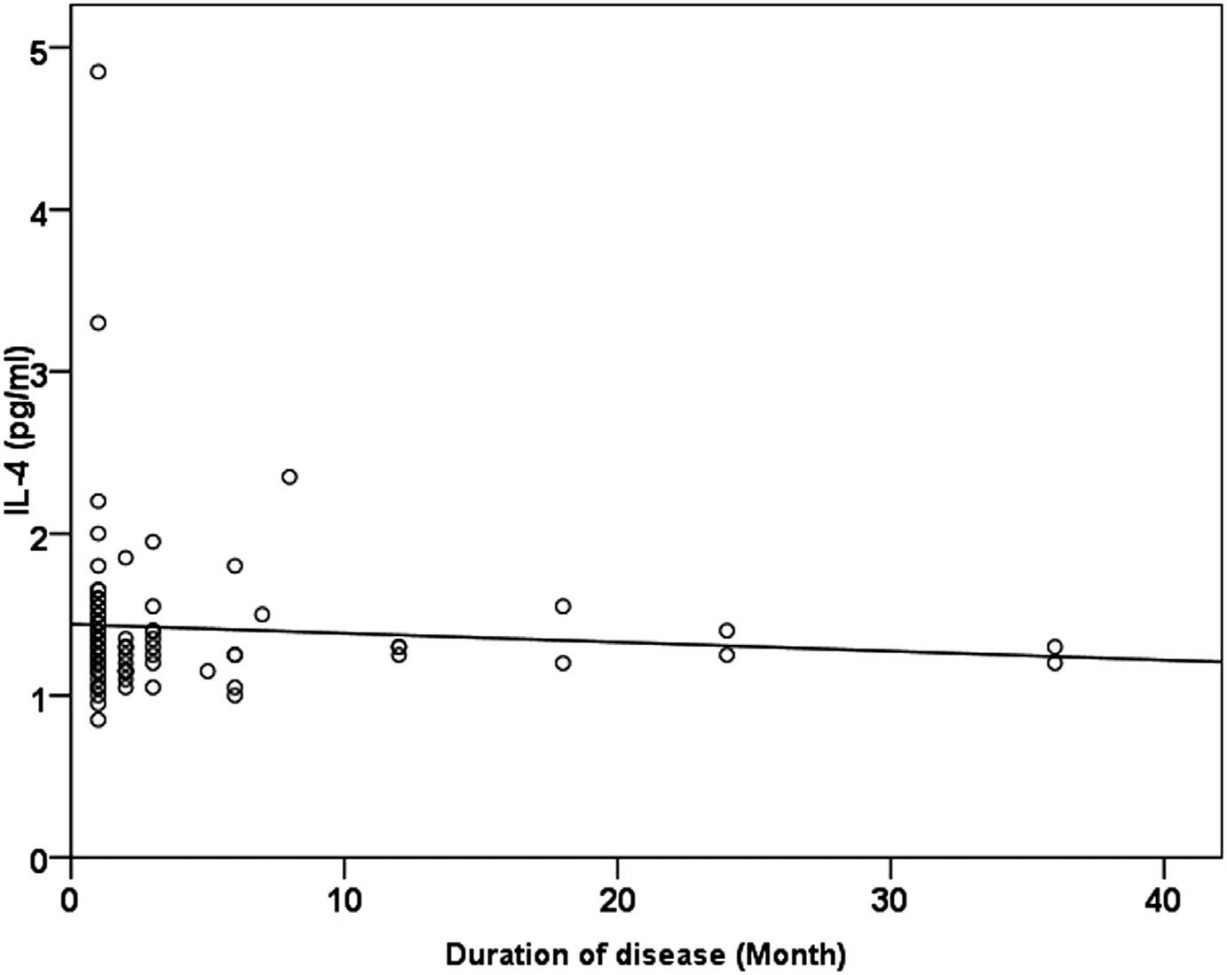
Distribution of serum levels of IL‐4, according to duration of brucellosis

The mean duration of brucellosis in the case group was 4.06 months (minimum, <1 month; maximum, 36 months). Based on the Spearman's correlation test, there was no significant correlation between the serum level of IL‐4 in patients with brucellosis and the duration of disease (Spearman's *ρ* = −.106, *P* = .34) (Figure 1). Also, according to Table 2 and the results of non‐parametric Kruskal‐Wallis test, there was no significant association between the mean serum level of IL‐4 and different titers of Wright and 2ME serological tests in patients with brucellosis.

**Table 2:** Serum levels of IL‐4 in patients with brucellosis, according to Wright and 2ME test titers

Serologic Test	Titer	Number	Mean of IL‐4 (pg/mL)	SD	*P* value
Wright	1/80	12	1.30	0.05	.459
	1/160	28	1.31	0.04	
	1/320	19	1.38	0.07	
	1/640	12	1.43	0.40	
	>1/640	12	1.43	0.40	
2ME	1/40	17	1.50	0.56	.669
	1/80	28	1.28	0.25	
	1/160	17	1.64	0.87	
	1/320	12	1.40	0.26	
	≥1/640	9	1.29	0.22	

## 4 DISCUSSION

Cytokines are proteins or glycoproteins, which act as adjuvant regulators of the immune system. IL‐4 is one of the most important cytokines in the body, which is secreted by white blood cells and contributes to inflammatory and immune responses.(19) The role and importance of IL‐4 in multiple diseases have been confirmed in various studies. These studies have indicated the decreased level of IL‐4 in patients with infectious mononucleosis, compared with healthy controls(10) and have reported the increased serum level of IL‐4 in patients with rheumatoid arthritis with and without interstitial lung disease (ILD).(20)

In addition, the increased serum level of IL‐4 has been reported in children with complicated *Plasmodium falciparum* infection*,* compared with uncomplicated children.(21) Moreover, the increased production of IL‐4 related to the severity of *falciparum* malaria parasitemia,(22) its protective role against Mycoplasma pulmonary diseases,(23) and its increased level associated with the increased severity of asthma(24) have been reported in the literatures.

In the present study, the level of IL‐4 in brucellosis patients was compared with healthy subjects and consistent with the results reported by Galanakis et al,(17) and the serum level of IL‐4 was significantly higher in brucellosis patients, compared with the healthy controls. On the other hand, a study by Demirdag et al(13) reported contradictory results. It should be noted that Demirdag(13) also found no significant difference in the level of IL‐4 before and after brucellosis treatment, which is contrary to the results reported by Reza et al, suggesting a significant reduction in IL‐4 following brucellosis treatment. However, in a study by Dashti et al,(25) there was no significant difference between the serum level of IL‐4 of patients with brucellosis and other febrile diseases. The cause of discrepancy between the results reported by Demirdag(13) and other researchers and also the non‐detectable serum level of IL‐4 in brucellosis patients evaluated in the study by Ahmed et al(16) may be due to the low sensitivity or high assay range of the used kit. In addition, racial differences and polymorphisms of genes in the pathway of IL‐4 should be considered. We used higher sample size than the study of Demirdag et al and High‐Sensitivity ELISA Kit for measuring of the serum level of IL‐4, in order to increase the power of the study.

Meanwhile, the results of the present study showed that in patients with brucellosis, there was no significant relationship between the serum level of IL‐4 and the patient's sex, age, duration of disease, and also serologic titers of Wright and 2ME tests; this finding indicates the independence of this cytokine from the mentioned factors. The independence of serum level of IL‐4 from the Wright and 2ME titers suggests the autonomy of cellular pathways of the immune system from humoral pathways, in exposure to the Brucella, as IL‐4 is considered a cellular immune system cytokine. Moreover, in the study by Reza et al,(18) the association of IL‐4 level with age and sex was not significant in brucellosis patients.

Due to the changes in serum level of IL‐4 following the onset of brucellosis, based on the results of the present study and previous researches, along with its changes after treatment of brucellosis as emphasized also in numerous previous studies,(13, 18) IL‐4 can be used to monitoring treatment process of brucellosis. For this purpose, larger studies are required in different populations.

## 5 CONCLUSION

In patients with brucellosis, regardless of age, sex, and duration of infection, the serum level of IL‐4 increased following the disease, which indicates the role of IL‐4 in the pathophysiology of disease and its close relationship with the activation of inflammation and immune system pathways in exposure to brucellosis. Therefore, this biomarker can be used if there are adequate facilities for monitoring the level of immunity and response to treatment.
